# Maternal immunisation with trivalent inactivated influenza vaccine for prevention of influenza in infants in Mali: a prospective, active-controlled, observer-blind, randomised phase 4 trial

**DOI:** 10.1016/S1473-3099(16)30054-8

**Published:** 2016-09

**Authors:** Milagritos D Tapia, Samba O Sow, Boubou Tamboura, Ibrahima Tégueté, Marcela F Pasetti, Mamoudou Kodio, Uma Onwuchekwa, Sharon M Tennant, William C Blackwelder, Flanon Coulibaly, Awa Traoré, Adama Mamby Keita, Fadima Cheick Haidara, Fatoumata Diallo, Moussa Doumbia, Doh Sanogo, Ellen DeMatt, Nicholas H Schluterman, Andrea Buchwald, Karen L Kotloff, Wilbur H Chen, Evan W Orenstein, Lauren A V Orenstein, Julie Villanueva, Joseph Bresee, John Treanor, Myron M Levine

**Affiliations:** a Center for Vaccine Development, University of Maryland School of Medicine, Baltimore, MD, USA; b Le Centre pour le Développement des Vaccins du Mali (CVD-Mali), Bamako, Mali; c Department of Obstetrics and Gynecology, Hôpital Gabriel Touré, Bamako, Mali; d Cooperative Studies Program Coordinating Center, Department of Veterans Affairs, Perry Point, MD, USA; e Department of Epidemiology, University of Maryland, Baltimore, MD, USA; f Emory University School of Medicine, Atlanta, GA, USA; g Department of Pediatrics, Children's Hospital of Philadelphia, Philadelphia PA, USA; h Department of Dermatology, University of Pennsylvania Hospital, Philadelphia PA, USA; i National Center for Immunization and Respiratory Diseases, Centers for Disease Control and Prevention, Atlanta, GA, USA; j Division of Infectious Diseases, Department of Medicine, University of Rochester School of Medicine and Dentistry, Rochester, NY, USA

## Abstract

**Background:**

Despite the heightened risk of serious influenza during infancy, vaccination is not recommended in infants younger than 6 months. We aimed to assess the safety, immunogenicity, and efficacy of maternal immunisation with trivalent inactivated influenza vaccine for protection of infants against a first episode of laboratory-confirmed influenza.

**Methods:**

We did this prospective, active-controlled, observer-blind, randomised phase 4 trial at six referral centres and community health centres in Bamako, Mali. Third-trimester pregnant women (≥28 weeks' gestation) were randomly assigned (1:1), via a computer-generated, centre-specific list with alternate block sizes of six or 12, to receive either trivalent inactivated influenza vaccine or quadrivalent meningococcal vaccine. Study personnel administering vaccines were not masked to treatment allocation, but allocation was concealed from clinicians, laboratory personnel, and participants. Infants were visited weekly until age 6 months to detect influenza-like illness; laboratory-confirmed influenza diagnosed with RT-PCR. We assessed two coprimary objectives: vaccine efficacy against laboratory-confirmed influenza in infants born to women immunised any time prepartum (intention-to-treat population), and vaccine efficacy in infants born to women immunised at least 14 days prepartum (per-protocol population). The primary outcome was the occurrence of a first case of laboratory-confirmed influenza by age 6 months. This trial is registered with ClinicalTrials.gov, number NCT01430689.

**Findings:**

We did this trial from Sept 12, 2011, to Jan 28, 2014. Between Sept 12, 2011, and April 18, 2013, we randomly assigned 4193 women to receive trivalent inactivated influenza vaccine (n=2108) or quadrivalent meningococcal vaccine (n=2085). There were 4105 livebirths; 1797 (87%) of 2064 infants in the trivalent inactivated influenza vaccine group and 1793 (88%) of 2041 infants in the quadrivalent meningococcal vaccine group were followed up until age 6 months. We recorded 5279 influenza-like illness episodes in 2789 (68%) infants, of which 131 (2%) episodes were laboratory-confirmed influenza. 129 (98%) cases of laboratory-confirmed influenza were first episodes (n=77 in the quadrivalent meningococcal vaccine group *vs* n=52 in the trivalent inactivated influenza vaccine group). In the intention-to-treat population, overall infant vaccine efficacy was 33·1% (95% CI 3·7–53·9); in the per-protocol population, vaccine efficacy was 37·3% (7·6–57·8). Vaccine efficacy remained robust during the first 4 months of follow-up (67·9% [95% CI 35·1–85·3] by intention to treat and 70·2% [35·7–87·6] by per protocol), before diminishing during the fifth month (57·3% [30·6–74·4] and 60·7 [33·8–77·5], respectively). Adverse event rates in women and infants were similar among groups. Pain at the injection site was more common in women given quadrivalent meningococcal vaccine than in those given trivalent inactivated influenza vaccine (n=253 *vs* n=132; p<0·0001), although 354 [92%] reactions were mild. Obstetrical and non-obstetrical serious adverse events were reported in 60 (3%) women in the quadrivalent meningococcal vaccine group and 61 (3%) women in the trivalent inactivated influenza vaccine group. Presumed neonatal infection was more common in infants in the trivalent inactivated influenza vaccine group than in those in the quadrivalent meningococcal vaccine group (n=60 *vs* n=37; p=0·02). No serious adverse events were related to vaccination.

**Interpretation:**

Vaccination of pregnant women with trivalent inactivated influenza vaccine in Mali—a poorly resourced country with high infant mortality—was technically and logistically feasible and protected infants from laboratory-confirmed influenza for 4 months. With adequate financing to procure the vaccine, implementation will parallel the access to antenatal care and immunisation coverage of pregnant women with tetanus toxoid.

**Funding:**

Bill & Melinda Gates Foundation.

**Research in context**

**Evidence before this study**

Immunisation of pregnant women against influenza has been common practice in industrialised nations since their vulnerability to severe disease and adverse outcomes was recognised. Nevertheless, this practice has not been adopted by low-income countries with constrained resources. When we undertook this trial, maternal immunisation as a strategy to prevent infant illness and avert associated morbidity and mortality was gaining traction. We searched PubMed between April 17, 1996, and March 8, 2016, for clinical trials with the terms “maternal influenza vaccination” and “maternal influenza immunization”. Our search yielded 36 publications. Two publications were of randomised clinical trials done in low-income and middle-income countries that measured the efficacy of maternal influenza vaccination in protection of infants. The first trial, done in Bangladesh, reported 63% efficacy in the reduction of laboratory-confirmed influenza in infants aged up to 24 weeks. Furthermore, infants born to women who received influenza vaccine were less likely to be small for gestational age and had a higher mean birthweight than did those born to women in the control group. The next trial was done in South Africa and reported 48·8% efficacy in infants aged up to 24 weeks; however, the other benefits to infants were not shown.

**Added value of this study**

Our trial represents the largest evaluation so far of maternal influenza vaccination as a strategy to prevent influenza in the youngest infants. Additionally, it is the first such study to be completed in west Africa, specifically Mali, one of the poorest countries in the world. Demonstrating the efficacy of maternal influenza vaccination in this setting is compelling. Moreover, establishing that efficacy is highest in the first 4 months of life (67·9%) is important as the duration of protection conferred through maternal vaccination and anticipated benefits are assessed. Finally, the absence of an effect on the incidence of low birthweight is consistent with findings shown in South Africa.

**Implications of all the available evidence**

Our study unequivocally demonstrates efficacy of maternal influenza vaccination against laboratory-confirmed influenza in infants and mothers. Moreover, there was high acceptability and logistical feasibility. However, our trial and that done in South Africa did not corroborate the previously reported benefits on neonatal outcomes. Moreover, because these trials were not designed to measure an effect on severe, deadly disease, there remains a notable gap when assessing the cost-effectiveness of this intervention. Although the success of maternal tetanus immunisation programmes suggests that implementation of influenza vaccination would also be successful, the related cost would need to be justified by the gains of the benefits afforded so that local policy makers and donors could invest their restricted funds.

## Introduction

Pregnant women and young infants are at increased risk of developing severe, complicated, and sometimes fatal influenza infection;^1^, ^2^, ^3^, ^4^ however, no influenza vaccines are approved for infants younger than 6 months.^5^, ^6^, ^7^ Maternal immunisation against influenza is a promising strategy to reduce disease in pregnant women and young infants.^8^, ^9^ Trials in Bangladesh^10^ and South Africa^11^ showed protection against laboratory-confirmed influenza in infants born to mothers who received trivalent inactivated influenza vaccine, but additional health benefits in those infants (eg, higher birthweight and reduced likelihood of being small for gestational age) have been inconsistent.^11^, ^12^ Remaining questions include more precise determination of the duration of protection for infants that can accrue from maternal immunisation,^8^ and the technical and logistical feasibility and effectiveness of implementation of programmes in resource-limited settings with high to moderate infant mortality rates.^13^

We aimed to address these questions in the course of a post-licensure clinical trial of the safety, immunogenicity, and efficacy of maternal influenza immunisation for prevention of influenza in infants younger than 6 months in Mali, west Africa—one of the world's least developed countries, with the world's seventh highest infant mortality rate.^14^, ^15^ Mali, nevertheless, maintains a vaunted Expanded Program on Immunization (EPI) that includes immunisation of pregnant women with tetanus toxoid and the introduction of five new EPI vaccines for infants since 2005.^16^, ^17^, ^18^ Introduction of additional vaccines for pregnant women, particularly trivalent inactivated influenza vaccine, the composition of which changes annually, would be challenging, but would make use of an existing effective vaccine delivery platform.

## Methods

### Study design and participants

We did this prospective, active-controlled, observer-blind, randomised phase 4 trial at six referral centres and community health centres in Bamako, Mali. In the year before starting the trial, influenza activity occurred from September to April, with peaks in October and February.

Third-trimester pregnant women (≥28 weeks' gestation based on last menstrual period, ultrasound, or uterine height) presenting to participating health centres for prenatal care were eligible for inclusion. Participants had to be able to understand and comply with planned study procedures, provide written informed consent before initiation of any study procedures, and intend to reside in the study area until their newborn infants were 6 months old. Participants could not be members of a household that already had a woman who was participating or had participated in this study. Other exclusion criteria were a history of severe reactions following previous immunisation with influenza or meningococcal vaccines; Guillain–Barré syndrome; known allergy or hypersensitivity to eggs, egg proteins, latex, diphtheria toxoid, or any other components of trivalent inactivated influenza vaccine (Vaxigrip) and quadrivalent meningococcal conjugate vaccine (Menactra); known chronic medical disorder that, in the judgment of the investigator, could compromise assessment of the study vaccine or put the participant at risk; known active infection with HIV, hepatitis B virus, or hepatitis C virus; complications with the ongoing pregnancy, including preterm labour (with cervical change), placental abruption, premature rupture of membranes, known major congenital anomaly, or pre-eclampsia; acute illness or an oral temperature greater than or equal to 37·8°C within 72 h of vaccination (resulted in a temporary delay of vaccination); receipt of any other vaccine, excluding tetanus toxoid, within 2 weeks (for inactivated vaccines) or 4 weeks (for live vaccines and meningococcal A conjugate vaccine) before vaccination in this study; receipt of immunoglobulins or any blood products within 30 days before administration of study vaccines; chronic administration of immunosuppressants or other immune-modifying drugs within 90 days before administration of study vaccines; or any disorder that, in the opinion of the investigator, might compromise the wellbeing of the participant or compliance with study procedures, or interfere with the assessment of study vaccines. We additionally excluded women who intended to travel out of the study area in the 40 days after delivery. Enrolment continued until the requisite number of laboratory-confirmed influenza cases was detected in infants born to vaccinated women.

Approval for the research was obtained from the University of Maryland, Baltimore Institutional Review Board; the ethics committee of the Faculté de Médecine, Pharmacie et Odonto-Stomatologie of Mali; and the Ministry of Health of Mali. Community sensitisation was achieved through community leaders, health centre representatives and community members who attended community-wide meetings. All participants provided informed consent. If the participant was illiterate, consent was obtained in the presence of a literate witness after listening to the audiotaped version of the consent form in Bambara, the local language.

### Randomisation and masking

Participants were randomly allocated (1:1), via a computer-generated, centre-specific list with alternate block sizes of six or 12, to receive trivalent inactivated influenza vaccine (Vaxigrip, Sanofi Pasteur, Lyon, France) or quadrivalent meningococcal conjugate vaccine (Menactra, Sanofi Pasteur, Lyon, France). At enrolment, consenting participants were assigned an identification number, which at vaccination was referenced on the randomisation list and the allocated treatment given. The identification numbers for ineligible participants or those who withdrew before vaccination were not reassigned.

Study personnel who administered study vaccines and were aware of treatment allocation had no contact with participants after vaccination and were instructed not to reveal the identity of the study vaccines either to participants or to personnel masked to treatment allocation. Although the syringes used to administer the vaccines were different in appearance, participants were instructed to look away from the vaccinator and were unaware of the assigned intervention.

### Procedures

Quadrivalent meningococcal conjugate vaccine, rather than placebo, was given to controls to provide a potential benefit for all participants in this poor, mostly illiterate, vulnerable population of pregnant women. Moreover, that vaccine was unlikely to interfere with the primary outcome of the trial, yet would provide protection against meningococcal disease. Although disease due to serogroup A has largely disappeared from the region, other serogroups continue to cause epidemics in Mali.

The composition of trivalent inactivated influenza vaccine, supplied in prefilled syringes, changed during the trial. From September, 2011, to November, 2012, A/California/7/2009(H1N1[pandemic]-like), A/Perth/16/2009(H3N2)-like, and B/Brisbane/60/2008-like (2011 northern hemisphere formulation and then 2012 southern hemisphere formulation) were administered. From December, 2012, to April, 2013, A/California/7/2009 (H1N1[pandemic]-like), A/Victoria/361/2011(H3N2)-like strain, and B/Wisconsin/1/2010-like (2012 northern hemisphere formulation) were administered. Quadrivalent meningococcal conjugate vaccine, composed of 4 μg each of *Neisseria meningitidis* serogroup A, C, Y, and W-135 polysaccharides conjugated to diphtheria toxoid protein, was supplied in single-dose vials. A single 0·5 mL dose of trivalent inactivated influenza vaccine or quadrivalent meningococcal conjugate vaccine was injected into the deltoid muscle. Study vaccines were stored in secure, temperature-monitored refrigerators or cold rooms at 2–8°C.

After vaccination, women were observed for 30 min. 7 days after vaccination, field personnel interviewed the women about any local and systemic reactions. 28 days after vaccination, participants were clinically evaluated. Additional visits to evaluate safety and immunogenicity in women and infants were done at delivery and when the infant was 3 months and 6 months old. Each evaluation included a physical examination and blood specimen collection. When available, the infant birth sample was cord blood; otherwise, the birth sample was collected within 7 days after birth. To determine gestational age at birth, the New Ballard Score was measured at delivery or within 7 days after birth.^19^ Serious adverse events were recorded throughout study participation.

Besides safety follow-up visits, from enrolment to when the infant reached age 6 months, field personnel undertook weekly visits to detect influenza-like illness and severe acute respiratory infection. During each visit, the participating woman and infant (if already born) had their temperatures measured and were examined for influenza-like illness; women were additionally examined for severe acute respiratory infection. When case definitions for either disease were met (appendix p 5), nasopharyngeal and oropharyngeal swabs and a malaria blood smear were obtained. If influenza was detected by RT-PCR, the case was deemed to be laboratory-confirmed influenza. Standard-of-care treatment was offered.

Because the primary objective was to measure the efficacy of maternal immunisation for prevention of laboratory-confirmed influenza in their infants younger than 6 months, women were withdrawn from weekly surveillance of influenza-like illness following stillbirth, fetal death, infant death, or other events that precluded infant surveillance. Nevertheless, safety follow-up of women continued until 6 months after delivery. Appendix p 6 describes methods for sample collection, RT-PCR to detect influenza virus, virus subtyping, and haemagglutination inhibition antibody measurement.

### Outcomes

We assessed two coprimary objectives: vaccine efficacy in infants born to women vaccinated any time prepartum (intention-to-treat analysis), and vaccine efficacy in infants born to women vaccinated at least 14 days prepartum (per-protocol analysis). The primary outcome was the occurrence of a first case of laboratory-confirmed influenza by age 6 months. Secondary outcomes were the occurrence of a first case of laboratory-confirmed influenza in women (prepartum and post partum); occurrence of a first case of febrile influenza-like illness by age 6 months in infants; occurrence of a first case of febrile influenza-like illness in women (prepartum and post partum); occurrence of local and systemic reactogenicity after injection, related serious adverse events for the entire follow-up period, and all pregnancy complications; levels of influenza virus antibodies by haemagglutination inhibition before and 4 weeks after vaccination, at delivery, and 3 and 6 months after delivery. Tertiary outcomes included the frequency of each influenza virus type circulating in the study population, the levels of maternally derived influenza virus haemagglutination inhibition antibodies present in infants at birth and at ages 3 and 6 months, birthweights of infants born at a health centre, and the occurrence of severe acute respiratory infection in pregnant women. Appendix p 6 lists additional outcomes not included in the manuscript.

### Statistical analysis

We calculated vaccine efficacy with the formula:

$$\text{VE}=\frac{1-\text{hP}}{\left(1-\text{P}\right)}$$

where VE is vaccine efficacy, h is the ratio of follow-up time up to age 6 months in infants born to recipients of quadrivalent meningococcal vaccine to the follow-up time in infants born to recipients of trivalent inactivated influenza vaccine, and P is the proportion of all cases of laboratory-confirmed influenza occurring by age 6 months in infants whose mothers received trivalent inactivated influenza vaccine. This calculation is equivalent to estimating vaccine efficacy as 1–R, where R is the ratio of laboratory-confirmed influenza incidence rates. We used the ratio of incidence rates, rather than the ratio of proportions of participants who had laboratory-confirmed influenza to account for infants lost to follow-up before age 6 months. We estimated vaccine efficacy in both the intention-to-treat and the per-protocol populations. Only infants' first laboratory-confirmed influenza episodes were counted. Follow-up time was time from birth to first case of laboratory-confirmed influenza, infants reaching age 6 months, or exiting the study. We calculated vaccine efficacy for each month of age (0–5 months) and cumulative to each month of age.

For safety outcomes, we used Fisher's exact tests and Student's *t* tests to compare the proportion of participants who had each event per vaccine group. We did time-to-event analysis using Cox proportional hazards regression with laboratory-confirmed influenza as the outcome to establish whether year of vaccination or timing of vaccination relative to delivery had an effect on efficacy. Birthweight analysis was limited to weights that were either 500 g and more or 5000 g and less. We compared birthweight between vaccine groups both overall and within influenza seasons, defined as months with higher-than-average rates of laboratory-confirmed illness (February to April, September to October).

Sample-size calculations were based on a comparison of the expected proportion, P, of all cases of laboratory-confirmed influenza that occurred by age 6 months in infants whose mothers received trivalent inactivated influenza vaccine to the null value, P_0_, using exact binomial calculations and assuming equal total follow-up time in each vaccine group (h=1). For the intention-to-treat analysis, we assumed a laboratory-confirmed influenza attack rate of 2·2% by age 6 months in infants born to recipients of quadrivalent meningococcal vaccine and a 55% reduction in the attack rate in infants of recipients of trivalent inactivated influenza vaccine, to 0·99%; therefore, p=0·31034 and P_0_=0·5. For a one-sided α of 0·025, 77 cases of laboratory-confirmed influenza were needed to ensure 90% power for the intention-to-treat analysis, implying a need for about 4828 participants. Allowing for a 10% loss to follow-up, the sample size calculated became about 5370 participants. For the per-protocol analysis, we assumed vaccine efficacy to be 60%—ie, a laboratory-confirmed influenza attack rate of 0·88% by age 6 months in infants born to recipients of trivalent inactivated influenza vaccine. To ensure 90% power to show a vaccine efficacy of more than 5%, 67 cases of laboratory-confirmed influenza were needed, implying a sample size of 4352 participants. Allowing for a 20% loss to follow-up, or for the mother receiving vaccine less than 14 days before delivery, the sample-size requirement became about 5440 participants.

Enrolment was closed once 77 cases of infant laboratory-confirmed influenza were recorded, but surveillance continued until the infants reached 6 months of age. A Data Safety Monitoring Board oversaw the study and reviewed data on a regular basis. We did analyses with Stata (version 14.0) and NCSS (version 10). We did power calculations with PASS (version 12). This trial is registered with ClinicalTrials.gov, number NCT01430689.

### Role of the funding source

The funder of the study had no role in the study design, data collection, data analysis, data interpretation, or writing of the report. The corresponding author had full access to all the data in the study and had final responsibility for the decision to submit for publication.

## Results

**Figure 1:**
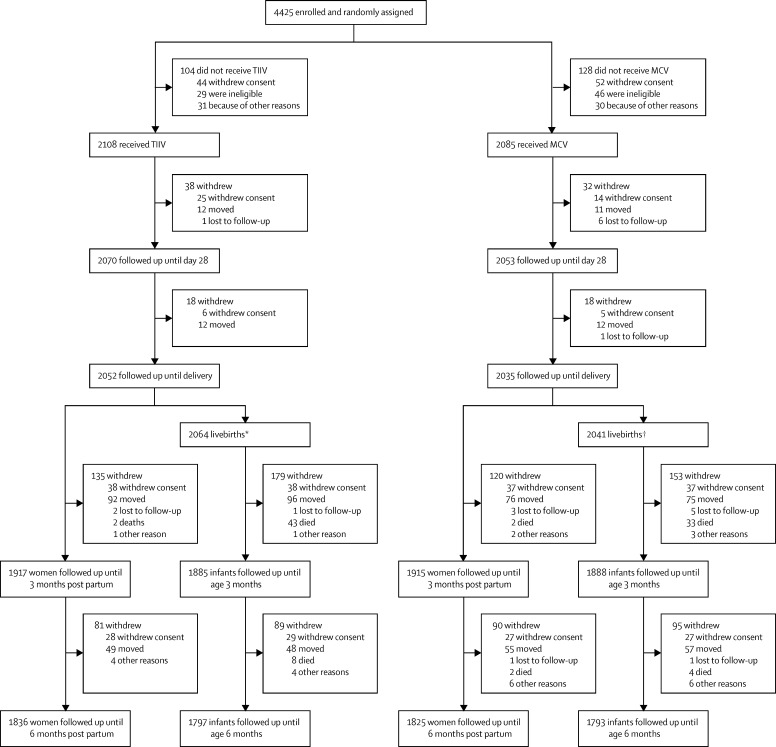
Trial profile TIIV=trivalent inactivated influenza vaccine. MCV=quadrivalent meningococcal conjugate vaccine. *1886 (91%) infants were born to women vaccinated with TIIV 14 or more days prepartum. †1869 (92%) infants were born to women vaccinated with MCV 14 or more days prepartum.

We did this trial from Sept 12, 2011, to Jan 28, 2014. Between Sept 12, 2011, and April 18, 2013, we randomly assigned 4193 women to receive trivalent inactivated influenza vaccine (n=2108) or quadrivalent meningococcal vaccine (n=2085; figure 1). Baseline characteristics were similar between groups (table 1). One (<1%) woman, who was inadvertently vaccinated twice (once with each vaccine), was followed up as part of her initial assignment group. 4087 (97%) women remained in the study until delivery; 3661 (87%) women were followed up until 6 months after delivery (figure 1). There were 4105 livebirths; 1797 (87%) of 2064 infants in the trivalent inactivated influenza vaccine group and 1793 (88%) of 2041 infants in the quadrivalent meningococcal vaccine group were followed up until age 6 months (figure 1). Due to political upheaval in Mali, study personnel were unable to do household visits for 1 week in March, 2012, and 2 weeks in May, 2012.

We recorded 5279 influenza-like illness episodes in 2789 infants younger than 6 months, of which 131 (2%) episodes were laboratory-confirmed influenza. 129 (98%) cases of laboratory-confirmed influenza were first episodes (n=77 in the quadrivalent meningococcal vaccine group *vs* n=52 in the trivalent inactivated influenza vaccine group). 116 (90%) first episodes of laboratory-confirmed influenza were in infants of women vaccinated at least 14 days prepartum. The 77 cases needed to complete vaccine efficacy analyses were reached by April 16, 2013; surveillance of post-partum women and their infants continued until infants reached age 6 months. One episode of laboratory-confirmed influenza was associated with malaria parasitaemia.

Overall infant vaccine efficacy was 33·1% (95% CI 3·7–53·9) in the intention-to-treat population, and 37·3% (7·6–57·8) in the per-protocol population (table 2). Notably, cumulative vaccine efficacy in infants in the intention-to-treat population was 67·9% in the first 4 months of follow-up, fell to 57·3% at the fifth month of surveillance, and dropped precipitously in the last month of follow-up, by which time protection was no longer evident (table 2). Cumulative vaccine efficacy in infants in the per-protocol population was 70·2% in the first 4 months of follow-up and 60·7% at the fifth month of surveillance (table 2). Within the trivalent inactivated influenza vaccine group, Cox regression analysis of the relative risk of laboratory-confirmed influenza showed that risk of influenza decreased when trivalent inactivated influenza vaccine had been given at least 15 days prepartum (p=0·02; appendix p 7). As long as the vaccine was given at least 15 days before delivery, no additional benefit was noted in women who had even longer intervals; women in neither the trivalent inactivated influenza vaccine group (Cox regression p=0·90) nor the quadrivalent meningococcal vaccine group (p=0·73) had a significant change in rates of laboratory-confirmed influenza as the time from delivery to vaccination increased above 14 days.

102 first episodes of laboratory-confirmed influenza in infants were due to influenza type A, including 41 H1N1, 59 H3N2, and two non-subtypeable viruses, and to 27 influenza B viruses (table 3). In the first 5 months of life, vaccine efficacy against influenza A was 64·5% overall, and 66·6% for H1N1 and 62·9% for H3N2 (table 3). No vaccine was effective against influenza type B in infants (table 3). In the first 6 months of life, no vaccine was effective against influenza-like illness (1% efficacy, 95% CI −7·0 to 8·5).

During the 29 months of surveillance of infants for laboratory-confirmed illness, the 131 circulating influenza viruses detected by RT-PCR changed over time and included 41 H1N1 viruses, 59 H3N2 viruses, two additional influenza A viruses that could not be subtyped, and 29 influenza B viruses (appendix p 8). We attempted to culture influenza virus from these 131 RT-PCR-positive infant clinical specimens to enable more definitive typing of viruses. Two contaminated samples could not be processed. Of the remaining 129 specimens, 65 grew influenza viruses, which were typed as A/California/7/2009(H1N1[pandemic]-like; n=32) detected from March, 2012, to October, 2013; A/Victoria/361/2011(H3N2; n=11) detected from September, 2012, to October, 2013; and B/Brisbane/03/2007 (Yamagata lineage; n=22) detected from November, 2012, to November, 2013. The H1N1 strains matched those in the trivalent inactivated influenza vaccine formulations; the H3N2 strains matched those in the second formulation and the B strains were of the same lineage as that within the second formulation.

From Sept 12, 2011, to Jan 28, 2014, we noted 1385 episodes of influenza-like illness in participating women; 52 (4%) episodes were laboratory-confirmed illness. 51 (96%) cases of laboratory-confirmed illness were first episodes. One episode was associated with malaria parasitaemia. Appendix p 9 summarises these cases. There were three episodes of severe acute respiratory infection, all of which were influenza negative. Overall vaccine efficacy was 70·3% (95% CI 42·2–85·8). Efficacy against influenza type A in women was 72·0% and against type B was 73·3% (table 3). Subtype-specific efficacy against H1N1 was 83·3% and could not be measured against H3N2 due to the few cases detected. Vaccine efficacy was 76·6% (95% CI 28·4–94·3) in pregnant women and 70·1% (28·0–89·1) in the post-partum period (appendix p 9). No vaccine was effective against maternal influenza-like illness (2·2% efficacy, 95% CI −10·5 to 13·5).

**Figure 2:**
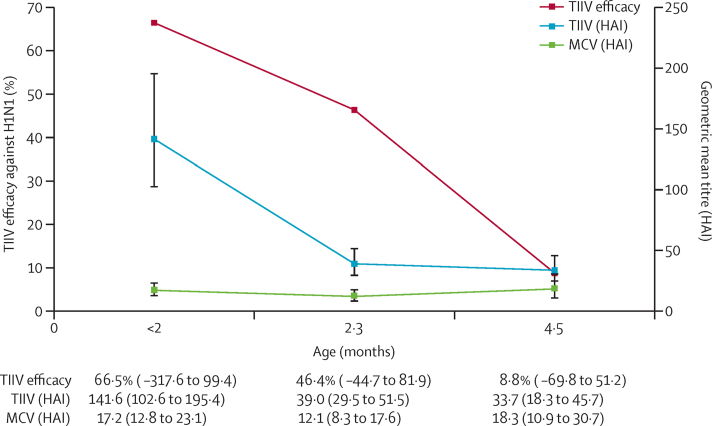
Vaccine efficacy and HAI antibody geometric mean titres in infants, by age and maternal vaccine group Error bars and data in parentheses show 95% CIs. TIIV=trivalent inactivated influenza vaccine. MCV=quadrivalent meningococcal conjugate vaccine. HAI=hemagglutination inhibition antibodies.

We measured haemagglutination inhibition antibody titres against influenza A/California/07/09 in 180 mother–infant pairs (figure 2). A subset of 43 pairs (plus one twin) constituted a nested case-control study in which we tested samples from 11 H1N1 cases (including a pair of twins) and 33 birthdate-matched controls (plus or minus 30 days). The remaining 137 pairs included 46 pairs with an infant with laboratory-confirmed illness, and 91 pairs with an infant who did not have laboratory-confirmed illness but might have had influenza-like illness; these pairs represented a convenience sample of participants who completed the study. By age 3 months, infant geometric mean titres (GMT) had decreased by more than 50%, although more infants in the trivalent inactivated influenza vaccine group had haemagglutination inhibition antibody titres of 40 or more (figure 2, appendix pp 10, 11). At age 6 months, haemagglutination inhibition antibody titres of 40 or more did not differ significantly between infants in either vaccine group, although maternal titres remained higher in the trivalent inactivated influenza vaccine group (appendix p 11). Of note, GMT increased in the quadrivalent meningococcal vaccine group at age 6 months, probably due to natural immunity acquired between ages 3 and 6 months. As haemagglutination inhibition antibody titres decreased with age in the trivalent inactivated influenza vaccine group, efficacy also decreased (figure 2).

The most frequently reported local and systemic reactions were pain at the injection site and febrile sensation (appendix p 12). Pain at the injection site was more commonly reported in women given quadrivalent meningococcal vaccine than in those given trivalent inactivated influenza vaccine (n=253 *vs* n=132; p<0·0001), although reactions were mostly mild (92%; appendix p 12). Rates of unrelated obstetrical and non-obstetrical serious adverse events in women were similar between groups (appendix p 13). The most commonly reported events were hypertensive disorders of pregnancy, which were equally common among both vaccine groups; 1% of participants in each group had pre-eclampsia (p=0·89; appendix p 13). No serious adverse event was related to study treatment. There were five (<1%) unrelated deaths among study participants (n=2 in the trivalent inactivated influenza vaccine group and n=3 in the quadrivalent meningococcal vaccine group); two (40%) patients died due to obstetrical complications and three (60%) patients died after the 42 day period after delivery.

Although rates of serious adverse events in infants were similar between groups, presumed neonatal infection was more common in infants in the trivalent inactivated influenza vaccine group than in those in the quadrivalent meningococcal vaccine group (n=60 *vs* n=37; p=0·02; appendix p 14). No serious adverse events in infants were related to maternal vaccination. 89 infants died: 52 (59%) infants in the trivalent inactivated influenza vaccine group and 37 (41%) infants in the quadrivalent meningococcal vaccine group (p=0·13; appendix p 14); no deaths were due to laboratory-confirmed influenza. Appendix p 15 summarises the timing and causes of death.

Per-protocol analysis of the number of infants with a Ballard score less than 33 yielded an overall prematurity rate of 1·8%, which did not correlate with rates measured using date of last menstrual period or results of first-trimester ultrasounds (appendix p 16). 358 (9%) liveborn infants were born at a low birthweight; there was no difference in birthweight between vaccine groups (p=0·20). Furthermore, there was no difference in birthweight among infants born during influenza season.

## Discussion

Here we report results of the largest randomised controlled trial so far of trivalent inactivated influenza vaccine in pregnant women, which was undertaken in Mali, where infant and maternal mortality rates are among the world's highest.^14^ Trivalent inactivated influenza vaccine elicited robust antibody responses and women and their infants were significantly protected against laboratory-confirmed influenza, corroborating results from Bangladesh (63% vaccine efficacy, 95% CI 5–85) and South Africa (48·8%, 11·6–70·4),^10^, ^11^ and supporting WHO recommendations that pregnant women should be the highest priority target for influenza vaccination.^20^ Because pregnant women and infants are at high risk for severe and fatal influenza illness even in affluent countries, our findings showing efficacy of maternal immunisation in severely resource-constrained Mali, one of the world's least developed countries, constitute encouraging new information.

Maternal immunisation with trivalent inactivated influenza vaccine provided robust protection to infants during the first 4 months of life. Thereafter, as haemagglutination inhibition antibody titres diminished, efficacy decreased and was no longer evident at month 6 of follow-up. These observations support the contention that transplacental maternal antibody protects infants against laboratory-confirmed influenza. Haemagglutination inhibition antibody kinetics resembled those reported in Bangladesh^21^ and South Africa,^22^ and align with findings from seroepidemiological studies^23^, ^24^ showing that by age 6 months most Malian infants no longer have protective titres of maternally derived measles and *Haemophilus influenzae* type b antibodies.

Influenza vaccine was well tolerated by pregnant women in our study, corroborating increasing evidence supporting the safety of trivalent inactivated influenza vaccine during pregnancy.^25^ The Bangladesh trial reported that infants born during the influenza season to women who received influenza vaccine had higher birthweights than did those born to women who received control vaccine during that period.^12^ In Mali and South Africa there was no beneficial effect of maternal immunisation with trivalent inactivated influenza vaccine on birthweight in infants born anytime during the study,^11^ including during influenza season. Exclusion of women with high-risk pregnancies from our study and inclusion of women late in pregnancy might have made it difficult to detect differences in birthweight due to maternal disorders. Moreover, infants born to women who had been in the study longer had higher birthweights than did those vaccinated closer to delivery, further decreasing the likelihood of detecting a difference between vaccine groups.

While demonstrating the efficacy and safety of maternal influenza immunisation, we were also able to address the technical and logistical feasibility of implementation of such a programme in Mali. The trial was well received by the community as the study team worked at local health centres alongside routine prenatal care (that included the administration of tetanus toxoid) to enrol more than 4000 women. The workflow pattern for administration of study vaccine paralleled that of tetanus toxoid administration and was well accepted by local providers. Nevertheless, remaining aspects, such as the availability of an appropriate vaccine, access to prenatal care, and cost, would affect implementation of a maternal influenza immunisation programme.

We noted seasonal influenza peaks with different influenza viruses from year to year, and these fluctuations affected vaccine efficacy. Notably, vaccinated mothers were significantly protected against influenza B, whereas infants were not. The probable explanation relates to when different B-virus lineages circulated. Cases of type B laboratory-confirmed influenza in infants born to mothers vaccinated with trivalent inactivated influenza vaccine were Yamagata lineage infections, whereas mothers had received B/Brisbane/60/2008 (Victoria lineage) vaccine. By the time infants born to women who received Yamagata lineage-containing vaccine (B/Wisconsin/1/2010) were exposed to influenza, little type B was circulating. This finding shows the complexity of vaccine selection and supports the use of quadrivalent influenza vaccines containing both type-B lineages. Timeliness of importation of newly formulated vaccine, promptness of initiation of vaccination of pregnant women and the types of circulating influenza viruses in relation to vaccine viruses, all affect vaccine efficacy. If maternal vaccination is to succeed in Mali, infants born in September to October will need to be protected. Because northern hemisphere influenza vaccine becomes available in August or September, immunisation will need to be implemented almost immediately upon vaccine importation. The second peak (February to April) does not present this logistical issue. An alternative strategy for countries with an influenza epidemiology similar to Mali is to use vaccine with an extended shelf-life throughout the year.^26^ This approach would allow immunisation of Malian pregnant women in the months leading up to the September to October peak before the newer formulation is available.

Another factor influencing the overall effect and sustainability of maternal immunisation in countries such as Mali is access to health-care interventions. The 2014 Demographic and Health Survey reported that whereas 95·2% of pregnant women in Bamako and 91·8% in other urban areas had at least one prenatal visit during their most recent pregnancy,^27^ this was true for only 69·3% of pregnant women in rural Mali. Barriers limiting access to health care in rural areas should be overcome to achieve high maternal immunisation coverage.

As other similarly low-resourced countries consider the implementation of a maternal influenza vaccination programme, the cost will also affect the feasibility. Since we did not observe an effect of trivalent inactivated influenza vaccine on birthweight, the cost-effectiveness of implementation of the vaccine in pregnancy to prevent infant influenza in Mali will hinge on prevention of severe illness and infant deaths. However, our study was not powered to measure the efficacy of trivalent inactivated influenza vaccine in the prevention of severe laboratory-confirmed influenza. Furthermore, because we visited households of study participants weekly, and intervened when illnesses were encountered by treating and referring participants earlier than they might have sought care in our absence, we probably interrupted progression of illness in many infants. Addressing this gap will require a different trial design and a larger sample size.

Our study unequivocally demonstrates efficacy of maternal immunisation against laboratory-confirmed influenza among infants and mothers, and shows high acceptability and logistical feasibility, thereby paving the way for a larger trial to assess prevention of severe laboratory-confirmed influenza leading to hospital admission in infants. Our findings support a vision that, in the future, developing countries might use the maternal immunisation platform to deliver vaccines to prevent respiratory syncytial virus,^28^ pertussis,^29^ influenza,^13^, ^26^ and tetanus.^30^

## Figures and Tables

**Table 1:** Baseline characteristics

		**TIIV group (n=2108)**	**MCV group (n=2085)**
Age (years)		24·7 (5·9)	24·7 (6·02)
Gravidity		3·2 (2·1)	3·3 (2·1)
Parity		2·1 (2·05)	2·1 (2·03)
Gestational age at enrolment (weeks)		32·6 (3·6)	32·6 (3·7)
Available method to estimate gestational age at enrolment			
	Early ultrasound (<15 weeks)	326 (15%)	322 (15%)
	Ultrasound after 15 weeks	667 (32%)	641 (31%)
	Date of last menstrual period	136 (6%)	134 (6%)
	Uterine height	979 (46%)	988 (47%)
Completed HIV testing		716 (34%)	696 (33%)
Time from vaccination to delivery (days)		53·7 (28·3)	53·3 (28·0)
Delivered at health centre		1966 (93·3%)	1988 (95·3%)
Delivery by cesarean section		128 (6%)	126 (6%)
Livebirths		2064 (98%)	2041 (98%)
Twin birth		36 (2%)	36 (2%)

TIIV=trivalent inactivated influenza vaccine. MCV=quadrivalent meningococcal conjugate vaccine.

**Table 2:** Maternal influenza vaccine efficacy against first episodes of laboratory-confirmed influenza in infants younger than 6 months born to women vaccinated at any time prepartum or 14 or more days prepartum

	**Born to women vaccinated at any time prepartum**									**Born to women vaccinated ≥14 days prepartum**								
	TIIV group (n=2064)				MCV group (n=2041)					TIIV group (n=1886)				MCV group (n=1869)				
	By month		Cumulative		By month		Cumulative			By month		Cumulative		By month		Cumulative		
	Days of follow-up	Cases*	Days of follow-up	Cases*	Days of follow-up	Cases*	Days of follow-up	Cases*	Cumulative vaccine efficacy (95% CI)	Days of follow-up	Cases*	Days of follow-up	Cases*	Days of follow-up	Cases*	Days of follow- up	Cases*	Cumulative vaccine efficacy (95% CI)
<1 month	61 254	0 (0·0)	61 254	0 (0·0)	60 719	6 (0·10)	60 719	6 (0·10)	100%(15·8 to 100)	55 981	0 (0·00)	55 981	0 (0·00)	55 602	5 (0·09)	55 602	5 (0·09)	100% (−8·4 to 100)
1 month	60 251	2 (0·03)	121 505	2 (0·02)	59 562	3 (0·05)	120 281	9 (0·07)	78·0% (−6·3 to 97·7)	55 181	2 (0·03)	111 162	2 (0·02)	54 637	3 (0·05)	110 239	8 (0·07)	75·2% (−24.2 to 97)
2 months	58 886	4 (0·07)	180 391	6 (0·03)	58 675	10 (0·17)	178 956	19 (0·11)	68·7%(18·4 to 89·8)	53 979	3 (0·06)	165 141	5 (0·03)	53 764	8 (0·15)	164 003	16 (0·10)	69·0%(11·.3 to 91·1)
3 months	57 468	5 (0·09)	2378 59	11 (0·05)	57 017	15 (0·26)	235 973	34 (0·14)	67·9%(35·1 to 85·3)	52 638	4 (0·08)	217 779	9 (0·04)	52 212	14 (0·27)	216 215	30 (0·14)	70·2%(35·7 to 87·6)
4 months	55 600	14 (0·25)	293 459	25 (0·09)	54 913	24 (0·44)	290 886	58 (0·20)	57·3%(30·6 to 74·4)	50 893	12 (0·24)	268 672	21 (0·08)	50 200	23 (0·46)	266 415	53 (0·20)	60·7%(33·8 to 77·5)
5 months	48 485	27 (0·56)	341 944	52 (0·15)	47 608	19 (0·40)	338 494	77 (0·23)	33·1%(3·7 to 53·9)	44 434	24 (0·54)	313 106	45 (0·14)	43 539	18 (0·41)	309 954	71 (0·23)	37·3%(7·6 to 57·8)

TIIV=trivalent inactivated influenza vaccine. MCV=quadrivalent meningococcal conjugate vaccine.

* Incidence per 1000 infant-days of follow-up.

**Table 3:** Number of cases of influenza and influenza vaccine efficacy against first episodes of laboratory-confirmed influenza by type in women and their infants up to 5 months of age

		**Women**				**Vaccine efficacy (95% CI)**	**Infants**				**Vaccine efficacy (95% CI)**
		TIIV group (n=2108)	Incidence per 1000 person-days of follow-up	MCV group (n=2085)	Incidence per 1000 person-days of follow-up		TIIV group (n=2064)	Incidence per 1000 person-days of follow-up	MCV group (n=2041)	Incidence per 1000 person-days of follow-up	
Type A		7	0·03	25	0·09	72·0% (35·2 to 87·9)	17	0·06	48	0·17	64·5% (38·3 to 79·6)
	H3N2	4	0·01	7	0·03	42·8% (−95·4 to 83·3)	10	0·04	27	0·10	62·9% (23·4 to 82·0)
	H1N1	3	0·01	18	0·06	83·3% (43·4 to 95·1)	7	0·02	21	0·07	66·6% (21·5 to 85·8)
Type B		4	0·01	15	0·05	73·3% (19·6 to 91·1)	8	0·03	10	0·04	19·9% (−103·0 to 68·4)

TIIV=trivalent inactivated influenza vaccine. MCV=quadrivalent meningococcal conjugate vaccine.
