# Supplementary material for: Maternal immunisation with trivalent inactivated influenza vaccine for prevention of influenza in infants in Mali: a prospective, active-controlled, observer-blind, randomised phase 4 trial
Source: Lancet Infect Dis. 2016 Sep;16(9):1026–35. doi: 10.1016/S1473-3099(16)30054-8 (PMC4985566; doi:10.1016/S1473-3099(16)30054-8)
Supplement: Supplementary appendix [file mmc1.pdf]

THE LANCET Infectious Diseases

Supplementary webappendix

This webappendix formed part of the original submission and has been peer reviewed. We post it as supplied by the authors.

Supplement to: Tapia MD, Sow SO, Tamboura B, et al. Maternal immunisation with trivalent inactivated influenza vaccine for prevention of influenza in infants in Mali: a prospective, active-controlled, observer-blind, randomised phase 4 trial. *Lancet Infect Dis* 2016; published online May 31. http://dx.doi.org/10.1016/S1473-3099(16)30054-8.

Supplementary Webappendix

This webappendix has been provided by authors to give the readers additional information about their work.

Supplement to: Tapia MD, et al. "**A prospective, active-controlled, observer-blind, randomized Phase 4 trial of the efficacy, safety and immunogenicity of trivalent inactivated influenza vaccine administered to third trimester pregnant women in Mali for the prevention of influenza in their infants up to 6 months of age**"

“A prospective, active-controlled, observer-blind, randomized Phase 4 trial of the efficacy, safety and immunogenicity of trivalent inactivated influenza vaccine administered to third trimester pregnant women in Mali for the prevention of influenza in their infants up to 6 months of age”

Milagritos D. Tapia,M.D., Samba O. Sow, M.D., Boubou Tamboura, Pharm.D., Ibrahima Teguete, M.D., Marcela F. Pasetti, Ph.D., Mamoudou Kodio, Pharm.D., Uma Onwuchekwa, B.S., Sharon M. Tennant, Ph.D., William C. Blackwelder, Ph.D., Flanon Coulibaly, M.D., Awa Traore, Pharm.D., Adama Mamby Keita, M.D., Fadima Cheick Haidara, M.D., Fatoumata Diallo, M.D., Moussa Doumbia, M.D., Doh Sanogo, M.D., Ellen DeMatt, M.A., Nicholas H. Schluterman, Ph.D., Andrea Buchwald, B.S., Karen L. Kotloff, M.D., Wilbur H. Chen, M.D., Evan W. Orenstein, M.D., Lauren A.V. Orenstein,M.D., Julie Villanueva, Ph.D., Joseph Bresee, M.D., John Treanor, M.D., Myron M Levine M.D.

Table of Contents

Study Team 4

Case Definition of Influenza-like Illness (ILI) in Infants 5

Case Definition of ILI and Severe Acute Respiratory Infection (SARI) in Women 5

Detection of Influenza Virus, Culture and Sub-typing 6

Serologic Responses 6

Additional outcomes not included in this manuscript 6

Table S1: Time to first LCI diagnosis by vaccine timing, birthweight, and date of vaccination 7

Figure S1: The monthly distribution of 129 LCI cases recorded in infants <6 months of age during the entire surveillance period, categorized by virus type and by vaccine received by the mother 8

Figure S2: Summary of 52 cases of laboratory-confirmed influenza among women observed throughout the study period – September 2011 to January 2014 9

Table S2: Number of cases of influenza observed and influenza vaccine efficacy against first episodes of laboratory-confirmed influenza in pregnant and post-partum women 9

Table S3: Geometric mean hemagglutination inhibition titers in study participants and their infants until 6 months of age, by visit and vaccine group 10

Table S4: Percent of study participants and their infants with HAI titers ≥40, by visit and vaccine group 11

Table S5: Local and systemic reactogenicity observed among 4187 women who completed at least the first 7 days of follow up 12

Table S6: Obstetrical and non-obstetrical serious adverse events observed among participating women at any time after vaccination until 6 months post-partum 13

Table S7: Serious adverse events (SAE) observed among infants born to participating women 14

Table S8: Timing and causes of death observed among the 89 infant deaths 15

Table S9: Ballard score and birth weight analysis among infants born to women vaccinated with either TIIV or MCV and born in the influenza season 16

References 17

Study Team

CVD-Mali
Clinical Team: Fadima Cheick Haidara, Fatoumata Diallo, Moussa Doumbia, Flanon Coulibaly, Ibrahima Tegueté, Doh Sanogo, Adama Mamby Keita, Evan Orenstein, Lauren Orenstein, Sidiki Koné, Bintou A. Tangara, Bréhima Diarra, Djeneba Traoré, Drissa Diarra, Abdoulaye Sidibé, Sékou Doumbia, Yacouba Diarra, Oumar Diakité, Sidi Diakité, Boubacar Bathily, Sibiry Samaté, Mamadou T. Keita, Yiriba Diarra, Aminata Traoré, Daouda Koné, Mariétou Samaké, Mama Camara, Awa Traoré, Moussa Sanogo, Oumar Mariko, Fatoumata Traoré, Aboubacar Samaké, Amadou Sagara, Fatoumata Maiga, Safiatou Coulibaly, Aissata Diarra, Tiècoura Bocoum, Balla Traoré, Karime Bayoko, Nana Gassama, Adama Diakité, Adama Coulibaly, Youssouf Diakité, Famakan Diango, Sinaly Dembele, Alassane Sangaré, Mama Mounkoro, Diakaridia Sidibé, Souleymane Diakité, Modibo Soumaré
Pharmacy Team: Mamoudou Kodio and Sekou Keita
Informatics and Support: Uma Onwuchekwa, Oualy Diawara, Moussa Traoré, Kounandji Diarra, Mahamane Djiteye, Ballan Sangaré
Laboratory Team: Boubou Tamboura, Awa Traoré, Abdoulaye Sangaré, Chaka Dit Tiédié Diallo, Aliou Touré, Oumarou A. Traore

CVD- University of Maryland
Laboratory Team: Mardi Reymann, Ifayet Mayo, Deanna Toema, Sofie Livio, Sunil Sen

Case Definition of Influenza-like Illness (ILI) in Infants

Either of the 2 following conditions reported by the caretaker or observed by a clinician:

- Fever without an apparent source, documented by a clinician's measurement to be an axillary temperature ≥38°C or maternal perception of fever and administration of antipyretic in previous 8 hours

  * No source means that there is no apparent cause for the fever such as soft tissue infection, although generalized symptoms such as irritability, loss of appetite, and/or lethargy may be present;

  OR

- Fever (as defined below)* plus acute respiratory infection.

  Acute respiratory infection is defined as ANY of the following on the same or consecutive days: runny nose, nasal congestion, cough, difficulty breathing, pus draining from ear or wheezing;

  PLUS

- > 7 days after last reported fever

Fever was defined as any of the following:

- Mother's perception that the infant had fever during the previous 24 hours
- Mother measured the infant's temperature as >38°C during the previous 24 hours
- Clinician or study staff measure the infant's temperature to be >38°C
- Maternal perception of fever and administration of antipyretic in previous 8 hours

Case Definition of ILI and Severe Acute Respiratory Infection (SARI) in Women

Women met ILI criteria if the following were observed by the examining physician or were part of clinical history:

- Onset of fever (oral temperature ≥38°C) < 7 days duration AND
- Cough or sore throat AND
- Absence of other diagnoses

  OR

- Onset of feverish feeling < 7 days duration AND
- Cough or sore throat or chest pain on breathing in AND
- Absence of other diagnoses

Women met SARI criteria if the following were observed by the examining physician or were part of clinical history:

- Sudden onset of fever over 38°C or perception of fever and self-administration of antipyretic in the previous 8 hours AND
- Cough or sore throat AND
- Shortness of breath or difficulty breathing
- Patient may or may not be hospitalized

Detection of Influenza Virus, Culture and Sub-typing

Using an ABI 7500 RT-PCR machine, RT-PCR kits from the Centers for Disease Control (CDC) were used to detect pandemic swine influenza, “seasonal” influenza A H3N2, “human” (pre-2009) H1N1 viruses and influenza B viruses. RNA extraction was performed following the recommended CDC method (QIAamp® viral RNA kit). RNA was tested immediately with the CDC real time RT-PCR protocol for detection and characterization of swine influenza. Analysis of data was performed using the software supplied with the Applied Biosystems™ real time PCR system ABI7500. Test runs in which positive and/or negative controls did not give the proper result were invalidated and the run was repeated. Positive samples were sent for viral culture and antigenic sub-typing according to standard methods.[1]

Serologic Responses

Seed viruses matching the vaccine strains were provided by the CDC Influenza Branch, and virus stocks were produced in embryonated hens’ eggs, as described.[2] Hemagglutination inhibition (HAI) antibody titers to specific vaccine components were measured by incubating serially-diluted serum samples (starting at 1:4) with 4 HA Units of each antigen and chicken erythrocytes, following standard techniques.[3] Sera were pre-treated with receptor destroying enzyme (Denka Seiken Co. Tokyo, Japan) to inactivate nonspecific inhibitors of viral hemagglutination.[4] HAI titers are calculated as the inverse of the highest dilution that inhibits hemagglutination.

Additional outcomes not included in this manuscript

An additional secondary outcome that will be presented in another paper includes the levels of maternal meningococcal antibodies (A, C, Y and W-135) measured by serum bactericidal assay, before and 4 weeks after vaccination, at delivery and 3 and 6 months after delivery. Additional tertiary outcomes that will be presented in another paper include: i) the occurrence of LCI among household contacts < 5 years of age; ii) the levels of maternally-derived serogroup-specific serum bactericidal antibodies (A, C, Y & W-135) at birth and 3 and 6 months of age; iii) the frequency of positive influenza by RT-PCR among samples collected from healthy infants aged 3 and 6 months (data are not yet analyzed); iv) the occurrence of pneumonia (as defined by the World Health Organization) in infants up to 6 months of age (data are not yet analyzed); v) the occurrence of meningococcal disease due to each of the vaccine serogroups (A, C, Y, W-135) in infants up to 6 months of age; vi) the cost of LCI and ILI in infants up to 6 months of age; vii) the cost of LCI and ILI in women

Table S1: **Time to first LCI diagnosis by vaccine timing, birthweight, and date of vaccination**

	Number of live-born babies	Number with LCI (%)	p-value (chi-square)	Hazard ratio[h]	95% Confidence interval	p-value (hazard ratio)
**Vaccine**						
MCV	2041	77 (3·8%)	0·02	Ref.		
TIIV	2064	52 (2.5%)		0·67	0·51-0·86	0·002
**Time from TIIV to Delivery[TIIV]**						
0-14 days	201	8 (4·0%)	0·17	Ref.		
15+ days	1862	44 (2.4%)		0·52	0·31-0·90	0·02
**Birth weight[TIIV]**						
<2500 g	191	4 (2·1%)	1[f]	Ref.		
2500+ g	1869	48 (2·6%)		1·07	0·52-2·21	0·85
Per 1000g increase*	N/A	N/A		1·21	0·79-1·87	0·38
**Birth weight[MCV]**						
<2500 g	167	7 (4·2%)	0·68[f]	Ref.		
2500+ g	1871	70 (3·7%)		0·81	0·46-1·41	0·46
Per 1000g increase**	N/A	N/A		1·04	0·72-1·50	0·84
**Date of vaccination[TIIV]**						
Sep 2011-July 2012	1117	29 (2·6%)	0·81	Ref.		
Aug 2012-Apr 2013	946	23 (2·4%)		1·21	0·80-1·84	0·36
**Date of vaccination**						
Interaction between date of vaccination and vaccine group	N/A	N/A		1·41	0·84-2·36	0·19

[f]Fisher's exact test. Other p-values in this column are from Pearson's chi-square test.
[TIIV]Among participants whose mothers were randomized to receive the influenza vaccine.
[MCV]Among participants whose mothers were randomized to receive the meningitis vaccine.
[h]Log-rank test, unadjusted for other variables
[c]Cox regression model with terms for vaccine assignment (not shown in table), date of vaccination (not shown in table), and interaction term between these two terms (shown in table)

Figure S1: **The monthly distribution of 129 LCI cases recorded in infants <6 months of age during the entire surveillance period, categorized by virus type and by vaccine received by the mother**

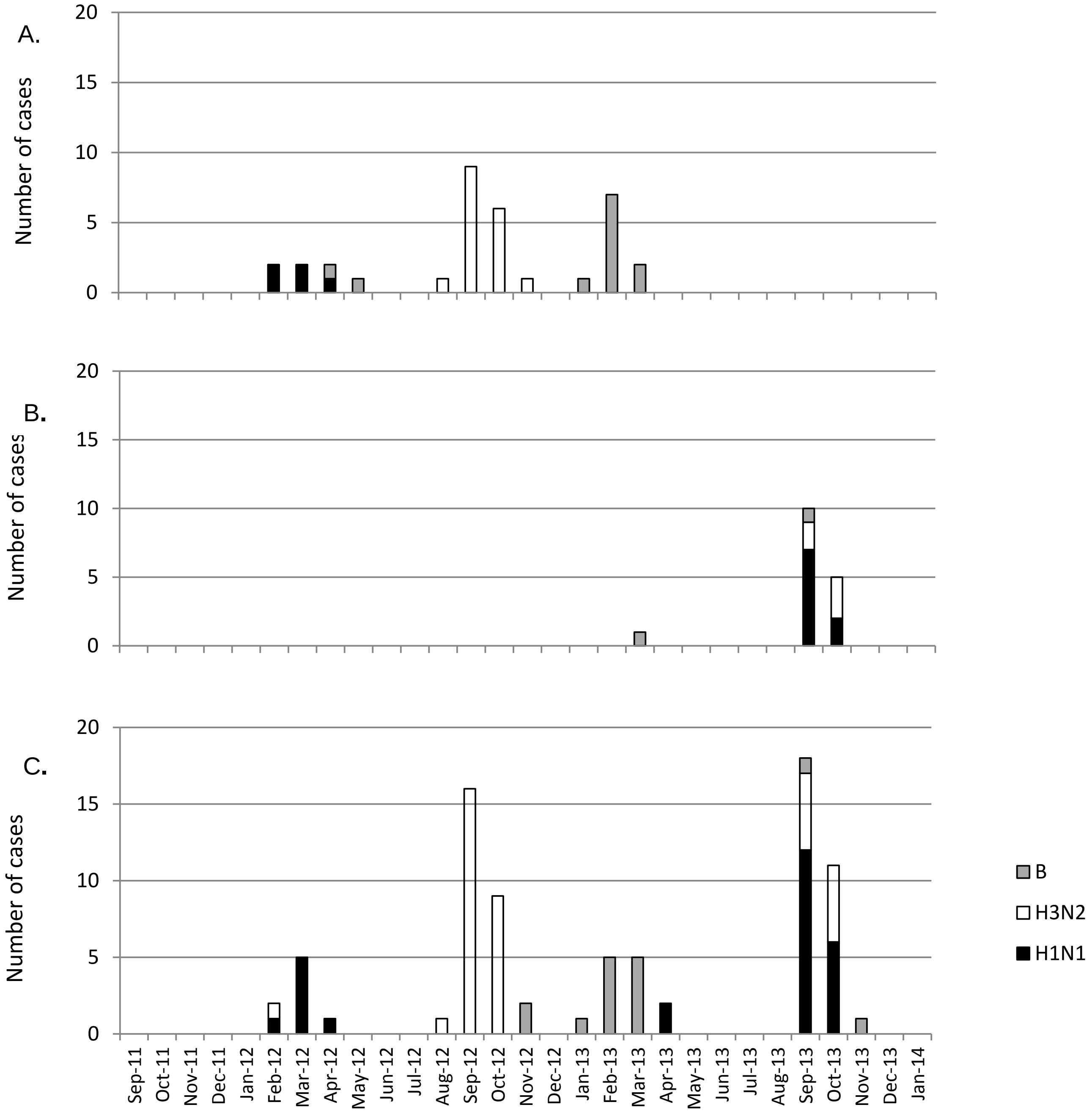

Panel A - A/California/7/2009(H1N1[pandemic]-like), A/Perth/16/2009(H3N2)-like and B/Brisbane/60/2008-like administered to pregnant women from September 2011-October 2012

Panel B - A/California/7/2009(H1N1[pandemic]-like), A/Victoria/361/2011(H3N2)-like and B/Wisconsin/1/2010-like administered to pregnant women from December 2012-April 2013

Panel C - Quadrivalent meningococcal conjugate vaccine administered to pregnant women September 2011-April 2013

Figure S2: **Summary of 52 cases of laboratory-confirmed influenza among women observed throughout the study period – September 2011 to January 2014**

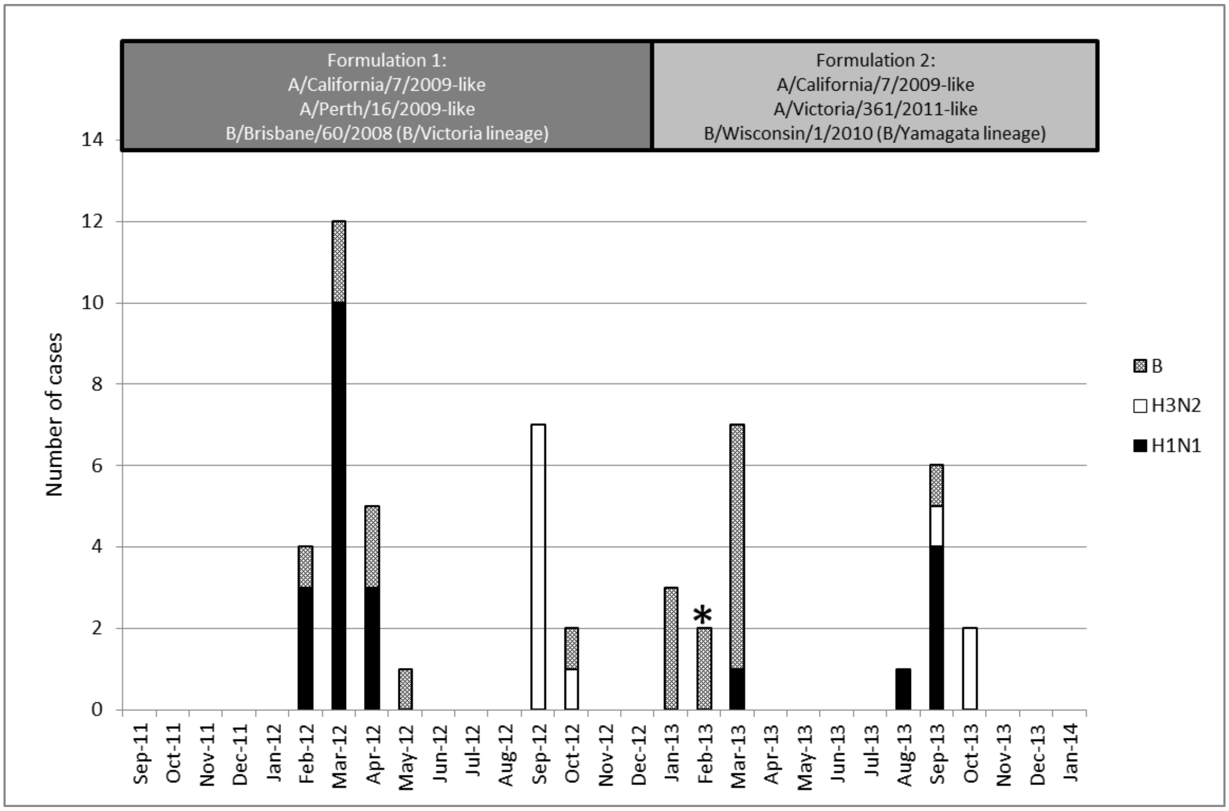

*Indicates that 1 of the 2 women who had LCI in February 2013 was randomized when Formulation 1 of TIIV was available and the other when Formulation 2 was available.

Figure S2 represents a summary of the 52 LCI cases that occurred among women in both vaccine groups over the entire surveillance period. Cases are classified according to influenza type. The bar at the top indicates the formulation of influenza vaccine that was available during the time that the women who experienced LCI were vaccinated. As of January 2013, all but one of the women who had LCI had been randomized when Formulation 2 was available.

Table S2: **Number of cases, incidence and influenza vaccine efficacy against first episodes of laboratory-confirmed influenza in pregnant and post-partum women**

	**TIIV N = 2108**		**MCV N = 2085**		**Vaccine Efficacy (95% CI)**
Period	Number of LCI cases	Incidence (per 1000 weeks observation)	Number of LCI cases	Incidence (per 1000 weeks observation)	
Pregnancy	4	0·30	17	1·30	76·6% (28·4 – 94·3)
Post-partum	7	0·17	23	0·58	70·1% (28·0 – 89·1)

Table S3: **Geometric mean hemagglutination inhibition titers in study participants and their infants until 6 months of age, by visit and vaccine group**

	TIIV				MCV				p-value[t]
**Mothers**	**n**	**GMT**	**95% CI**		**n**	**GMT**	**95% CI**		
Baseline	104	20·9	16·5	26·5	76	17·4	13·0	23·2	0·32
28 days post vaccination	88	311·7	231·0	420·6	58	17·6	13·0	23·9	<0·0001
Delivery	96	183·7	135·0	249·8	68	18·3	13·5	24·7	<0·0001
3 months after delivery	71	154·1	111·1	213·7	45	27·9	18·6	41·8	<0·0001
6 months after delivery	66	157·9	112·8	221·2	46	39·5	26·3	59·4	<0·0001
**Infants**									
Birth	96	141··6	102·6	195·4	67	17·2	12·8	23·1	<0·0001
3 months of age	70	39·0	29·5	51·5	45	12·1	8·3	17·6	<0·0001
6 months of age	67	33··7	24·8	45·7	46	18·3	10·9	30·7	0·03

[t]Student's t-test, comparing log_2-tranformed mean HAI titers between vaccine groups.

GMT: geometric mean titer.

CI: confidence interval.

***Table S4:* Percent of study participants and their infants with HAI titers ≥40, by visit and vaccine group**

	**TIIV**			**MCV**			**p-value[x]**
	**n ≥40**	**n**	**% ≥40**	**n ≥40**	**n**	**% ≥40**	
Baseline	27	104	26%	19	76	25%	0·88
28 days post vaccination	82	88	93%	14	58	24%	<0·0001
Delivery	83	96	86%	19	68	28%	<0·0001
3 months after delivery	60	71	85%	16	45	36%	<0·0001
6 months after delivery	53	66	80%	22	46	48%	<0·0001
**Infants**							
Birth	76	96	79%	19	67	28%	<0·0001
3 months of age	29	70	41%	7	45	16%	0·004
6 months of age	30	67	45%	13	46	28%	0·08

[x]Pearson's chi-square test, comparing proportion of HAI titers >40 between vaccine groups.

Table S5: **Local and systemic reactogenicity observed among 4187 women who completed at least the first 7 days of follow up**

		**TIIV**<br>**N = 2105**	**MCV**<br>**N = 2082**	**p-value**
	**Severity**	**N (%)**	**N (%)**	
**Local Reactogenicity**				
Pain	Mild	126 (3·0%)	228 (5·47%)	<0·0001
	Moderate	5 (0·12%)	23 (0·55%)	<0·0001
	Severe	1 (0·02%)	2 (0·05%)	1
Redness	Mild	6 (0·14%)	11 (0·26%)	0·23
	Moderate	0 (0·%0)	0 (0·0%)	1
	Severe	0 (0·0%)	1 (0·0%)	1
Swelling	Mild	12 (0·29%)	31 (0·74%)	0·003
	Moderate	1 (0·0%2)	2 (0·05%)	1
	Severe	0 (0·0%)	1 (0·02%)	1
≥ 1 local reaction		140 (6·65%)	258 (12·4%)	<0·0001
**Systemic Reactogenicity**				
Fatigue	Mild	8 (0·19%)	17 (0·4%1)	0·07
	Moderate	1 (0·02%)	1 (0·02%)	1
	Severe	0 (0·0%)	0 (0·0%)	1
Febrile sensation	Mild	41 (0·98%)	53 (1·27%)	0·21
	Moderate	1 (0·02%)	3 (0·07%)	0·37
	Severe	1 (0·02%)	0 (0·0%)	1
Headache	Mild	34 (0·82%)	46 (1·1%)	0·18
	Moderate	4 (0·10%)	4 (0·1%)	1
	Severe	1 (0·02%)	0 (0·0%)	1
Myalgia	Mild	4 (0·10%)	16 (0·38%)	0·007
	Moderate	1 (0·02%)	2 (0·05%)	0·62
	Severe	0 (0·0%)	0 (0·0%)	1
≥ 1 systemic reaction		73 (3·47%)	113 (5·43%)	0·002

Table S6: **Obstetrical and non-obstetrical serious adverse events observed among participating women at any time after vaccination until 6 months post-partum**

**Event**	**TIIV**<br>**N = 2108**<br>**N (%)**	**MCV**<br>**N = 2085**<br>**N (%)**	**p-value**
Gestational hypertension	4 (0·2%)	2 (0·1%)	0·69
Pre-eclampsia	26 (1·2%)	24 (1·2%)	0·89
Eclampsia	1 (0·1%)	6* (0·3%)	0·07
Chorioamnionitis	2 (0·1%)	1 (0·1%)	1
Premature rupture of membranes	3 (0·1%)	4 (0·2%)	1
Placenta previa	3 (0·1%)	6 (0·3%)	0·34
Placental abruption	6 (0·3%)	4 (0·2%)	0·75
Uterine rupture	2 (0·1%)	2 (0·1%)	1
Post-partum hemorrhage	2 (0·1%)	3 (0·1%)	1
Separation of pubic symphysis	1 (0·1%)	0 (0%)	1
HIV infection diagnosed post-vaccination**	1 (0·1%)	6 (0·3%)	1
Serious infection in pregnancy[#]	9 (0·4%)	3 (0·1%)	0·07
Peritonitis	1* (0·1%)	0 (0)	1
**Events unrelated to pregnancy (occurred > 42 days post-partum)**			
Cardiomyopathy	1 (0·1)	1 (0·1)	1
Fracture of right leg	1 (0·1)	0 (0)	1
Difficulty walking	0 (0)	1 (0·1)	1
Extra-pulmonary tuberculosis	0 (0)	1 (0·1)	1
Cervical fracture post trauma	0 (0)	1* (0·1)	1
Electrocution	0 (0)	1* (0·1)	1
Sudden death, likely of cardiac origin	1* (0·1)	0 (0%)	1

* 1 fatal case included.

** These diagnoses were unknown to the participant prior to enrollment.

[#] These episodes include cases of hospitalized malaria (7), respiratory infections (2), pyelonephritis (2) and unspecified post-partum infection (1).

Table S7: **Serious adverse events (SAE) observed among infants born to participating women**

**Event[§]**	**TIIV N = 2064***	**MCV N = 2041***	**P-value**
	**N (%)[#]**	**N (%)[#]**	
Stillbirth	24 (1·2%)**	30 (1·5%)	0·41
Major congenital malformation	6 (0·3%)	4 (0·2%)	0·75
Meconium aspiration syndrome	1 (0·1%)	1 (0·1%)	1
Presumed/ Neonatal infection	60 (2·9%)	37 (1·8%)	0·02
Perinatal asphyxia	26 (1·3%)	20 (1·0%)	0·46
Respiratory infection	29 (1·4%)	20 (1·0%)	0·25
Malaria	4 (0·2%)	3 (0·1%)	1
Meningitis (including 3 pneumococcal)	2 (0·1%)	4 (0·2%)	0·45
Low birthweight/ Small for gestational age	5 (0·2%)	7 (0·3%)	0·58
Gastrointestinal infection	3 (0·1%)	2 (0·1%)	1
Unspecified infection	2 (0·1%)	1 (0·1%)	1
Microbiologically-confirmed bacteremia	2 (0·1%)	1 (0·1%)	1
Abdominal distension/ obstruction	1 (0·1%)	4 (0·2%)	0·22
Sudden infant death syndrome	2 (0·1%)	2 (0·1%)	1
Other	6 (0·2%)	2 (0·1%)	0·29
Infant deaths	52 (2·5%)	37 (1·8%)	0·13

[§] Events are not mutually exclusive.

* Number of livebirths per vaccine group.

** Stillbirth rate is calculated among all births.

[#] Percentage of live births except where indicated otherwise.

Table S8: **Timing and causes of death observed among the 89 infant deaths**

**Category of event**	**TIIV**<br>**N = 52**			**MCV**<br>**N = 37**		
**Age of death**	**<7days**	**7-28 days**	**>28 days**	**<7days**	**7-28 days**	**>28 days**
Congenital anomaly	2	0	2	2	1	0
Infection*	4	1	12	3	5	6
Perinatal asphyxia or meconium aspiration syndrome	17	0	0	12	0	0
Prematurity	6	2	0	5	0	0
Other	0	2	1	0	0	1
Unknown	0	1	2	1	0	1
This category includes 4 episodes of gastroenteritis, 3 of malaria, 3 of meningitis, 11 of unknown neonatal infection, 1 of oral candidiasis, 8 of pneumonia and 1 of pseudomonas bacteremia.						

Table S9: **Ballard score and birth weight analysis among infants born to women vaccinated with either TIIV or MCV and born in the influenza season**

Preterm infant, by vaccine assignment	Total	TIIV	MCV	P-value	Correlation with Ballard**
Ultrasound in first trimester, available for 551 participants	67/551 (12·2%)	30/271 (11·1%)	37/280 (13·2%)	0·44	0·41
Date of last menstrual period, available for 207 participants	32/207 (15·5%)	17/109 (15·6%)	15/98 (15·3%)	0·95	0·23

Birthweight of infants, at time of live birth, by vaccine assignment	Total	TIIV N = 2063	MCV N = 2041	P-value**
Low birthweight (<2500 g)				
All live births	357 (8·7%)	191 (9·3%)	166 (8·2%)	0·20
Births during peak influenza season*	167 (8·9%)	90 (9·6%)	77 (8·2%)	0·29
Birthweight – mean g (SD)				
All live births	3016 (458)	3017 (472)	3015 (444)	0·91
Births during peak influenza season*	3007 (460)	3013 (483)	3002 (437)	0·61

Birthweight of infants, at time of live birth, by vaccine timing and assignment	Total	Time from vaccination to delivery		
		0-14 days	15+ days	P-value***
Birthweight – mean g (SD)				
Assigned to TIIV	3017 (472)	2918 (496) [N=201]	3027 (468) [N=1862]	0·002
Assigned to MCV	3015 (444)	2948 (449) [N=188]	3022 (443) [N=1852]	0·03

* Peak influenza season was September 1-October 31 and February 1-April 30 (n=942 infants in TIIV group and 942 infants in MCV group)

**p-values comparing birthweights between TIIV and MCV groups, by Student's t-tests.

***p-values comparing birthweights within each vaccine group between infants whose mothers were vaccinated 0-14 and 15+ days before delivery, by Student's t-tests.

References

1. Durviaux S, Treanor J, Beran J, et al. Genetic and antigenic typing of seasonal influenza virus breakthrough cases from a 2008-2009 vaccine efficacy trial. Clin Vaccine Immunol 2014;21:271-9.

2. Response WDoCDSa. WHO Manual on Animal Influenza Diagnosis and Surveillance 2002. Report No·: WHO/CDS/CSRN/NCS/2002.5 Rev·1.

3. EaWPHSCfDC USDoH. The hemagglutination inhibition test for influenza viruses· Atlanta, GA 1975.

4. Shortridge KF, Lansdell A. Serum inhibitors of A 2 -Hong Kong influenza virus haemagglutination. Microbios 1972;6:213-9.
